# Characterization and Multiscale Modeling of the Mechanical Properties for FDM-Printed Copper-Reinforced PLA Composites

**DOI:** 10.3390/polym14173512

**Published:** 2022-08-26

**Authors:** Arda Özen, Gregor Ganzosch, Christina Völlmecke, Dietmar Auhl

**Affiliations:** 1 Chair of Polymer Materials Science and Technologies, Institute of Material Science and Technology, Technische Universität Berlin, Ernst-Reuter-Platz 1, 10587 Berlin, Germany; 2 Chair of Continuum Mechanics and Materials Theory, Institute of Mechanics, Technische Universität Berlin, Einsteinufer 5, 10587 Berlin, Germany; 3 Stability and Failure of Functionally Optimized Structures Group, Institute of Mechanics, Technische Universität Berlin, Einsteinufer 5, 10587 Berlin, Germany

**Keywords:** additive manufacturing, multiscale homogenization, polymer composites, finite element method, fused deposition modeling (FDM), machine learning, digital image correlation

## Abstract

Additive manufacturing is an emerging technology and provides high design flexibility to customers. Fused deposition modeling (FDM) is an economical and promising additive manufacturing method. Due to its many advantages, FDM received great attention in recent years, and comprehensive studies are being undertaken to investigate the properties of FDM-printed polymers and polymer composites. As a result of the manufacturing technology employed in FDM, inner structures are changed with different process parameters, and thus, anisotropic properties are observed. Moreover, composite filaments such as particle- or fiber-reinforced polymers already have anisotropy before FDM printing. In this study, we investigate the effect of different process parameters, namely layer thickness and raster width on FDM-printed copper-reinforced poly(lactic acid) (PLA). Mechanical characterizations with a high-resolution camera are carried out for analyzing the deformation behaviors. Optical microscopy characterizations are performed to observe the mesostructural changes with various process parameters. Scanning electron microscopy (SEM) and an energy-dispersive X-ray spectroscopy (EDS) analysis are conducted for investigating the microstructure, specifically, copper particles in the PLA matrix. A 2D digital image correlation code with a machine learning algorithm is applied to the optical characterization and SEM-EDS images. In this way, micro- and mesostructural features, as well as the porosity ratios of the specimens are investigated. We prepare the multiscale homogenization by finite element method (FEM) simulations to capture the material’s response, both on a microscale and a mesoscale. We determined that the mesostructure and, thereby, the mechanical properties are significantly changed with the aforementioned process parameters. A lower layer thickness and a greater raster width led to a higher elasticity modulus and ultimate tensile strength (UTS). The optical microscopy analysis verified this statement: Decreasing the layer thickness and increasing the raster width result in larger contact lines between adjacent layers and, hence, lower porosity on the mesoscale. Realistic CAD images were prepared regarding the mesostructural differences and porosity ratios. Ultimately, all these changes are accurately modeled with mesoscale and multiscale simulations. The simulation results are validated by laboratory experiments.

## 1. Introduction

Additive manufactured components are created in an additive fashion [1,2,3] instead of subtractive manufacturing [4]. This is an increasingly employed production technique [4,5,6,7,8] and popularly known as 3D printing [2]. It provides high design flexibility and cost-effective solutions for designers [9]. CAD models of desired shapes are processed and converted to gcodes (generation codes) in slicer programs in order to supply the necessary process information, such as the layer thickness, temperature, printing speed, etc. [10]. Additive manufacturing methods have significant potential impact on the industry and considerable advantages over traditional production roots. These can be briefly summarized as industrial efficiency and customization, on-demand and decentralized manufacturing, printing complete systems, increased supply chain proficiency, and sustainable manufacturing solutions [11]. One of the significant improvements is achieved by General Electric (GE) through new additive manufacturing capabilities. A new fuel nozzle for LEAP engines (Leading Edge Aviation Propulsion) has been developed, which is relatively much stronger and is 25% more lightweight than commercial ones. These parts are designed to achieve the best flow geometry and enhance efficiency. In addition, design freedom and customization of additive manufactured parts lead to more straightforward upgrading to the latest design and provide quick maintenance possibilities. For instance, Siemens PGS (Power Generation Services) redesigned and developed burner tips through direct metal laser sintering (DMLS), which makes repairing ten times faster and reduces waste [12]. According to [13], the additive manufacturing categories are sheet lamination [14,15], material jetting [16,17], binder jetting [18,19], powder-bed fusion [20,21], vat photopolymerization [22,23], directed energy deposition [24,25], and materials extrusion [26,27,28].

Fused deposition modeling (FDM) falls into the materials extrusion category [29]. It was developed in the 1980s [30]. The low equipment cost and design flexibility by its layered production process [10] are the main advantages of this method. Polymers in a filament form are heated over molten state in a nozzle and deposited onto the printing bed. Highly anisotropic properties occur due to the process technique employed in FDM [31,32]. The process [10,33,34,35] and materials parameters [36] greatly affect the quality and the mechanical properties of the final products [10,33]. FDM has a critical advantage for fabricating parts by composites. For instance, FDM printing of metal-reinforced composites requires much lower energies than standard metal additive manufacturing methods [37]. The FDM process is optimized for the printing of Ti-6Al-4V composites [38], and their properties are tried to be estimated by using analytical [37] and numerical methods [39].

The material responses under deformation are modeled through constitutive equations, also called the material models. In additive manufacturing, there is an additional inner structural effect due to its layer-by-layer process method, which is extensively studied in [33]. The finite element method (FEM) is one of the most common techniques in solid bodies for computing the structural response under deformation. In order to simulate the mechanical response of FDM polymers by the FEM accurately, we investigate the material models with the characteristics of FDM [33,40,41,42,43].

In this paper, we experimentally examine the effect of the raster width and layer thickness in FDM-printed copper-reinforced poly(lactic acid) (PLA) composites. Tensile specimens are prepared with three different raster widths (0.4, 0.8, and 0.96 mm) and two different layer thicknesses (0.2 and 0.3 mm). We present how the raster width and layer thickness change the porosity in the inner structures. The porosity is suggested herein as a measure of the macroscopic material’s response. Specimens from each group were examined under the optical microscope. For measuring the porosity ratio, a machine learning code is implemented. More information about the machine learning code development for porosity measurements can be found in [33].

Moreover, we prepare the digital twin of these experiments by multiscale computations using the FEM in two steps.

- On the microscale, we employed asymptotic homogenization with periodic boundary conditions. The code was developed by the open-source FEniCS computing platform. For more information, we refer to [44]. The CAD models of the representative volume elements (RVEs) were prepared in the open-source Salome 9.3.0 platform.
- On the mesoscale, elasto-static uniaxial tensile tests were computed by FEniCS. A direct homogenization approach for different loading cases was employed, and thereby, the elasticity parameters were calculated. For more information, we refer to [33]. The CAD models of the mesostructures were prepared after the microscopy–porosity analysis in Salome 9.3.0.

As a result, we qualitatively and quantitatively investigate the effects of raster width and layer thickness selection on the mesoscale and the effect of copper particles on the microscale.

This paper is structured by explaining the process parameters of FDM and the used material of copper-reinforced PLA, as well as the experimental setup in Section 2. Then, more experimental details such as uniaxial tensile tests, optical investigations, and the results are given in Section 3. Finally, the multiscale simulation is explained in Section 4, followed by conclusive remarks.

## 2. Materials and Methods

### 2.1. Production of Specimens via Additive Manufacturing

Copper-reinforced PLA filaments with 2.85 mm diameter were purchased from PrimaCreator (Malmö, Sweden). The specimens were produced by an FDM-type 3D printer, namely Ultimaker 3 Extended (Ultimaker B.V., Geldermalsen, Netherlands). CAD geometries of tensile specimens were drawn in the Salome 9.3 and exported as *.stl* files. The desired process parameters such as slicing speed, temperature, layer thickness, etc., were applied by the Ultimaker Cura 4.6.0 (Ultimaker B.V., Geldermalsen, The Netherlands) in G-codes. The process parameters are provided in Table 1. For slicing strategies and reducing trial-and-error iterations, thereby a sustainable production, we refer to [10].

**Figure 1:**
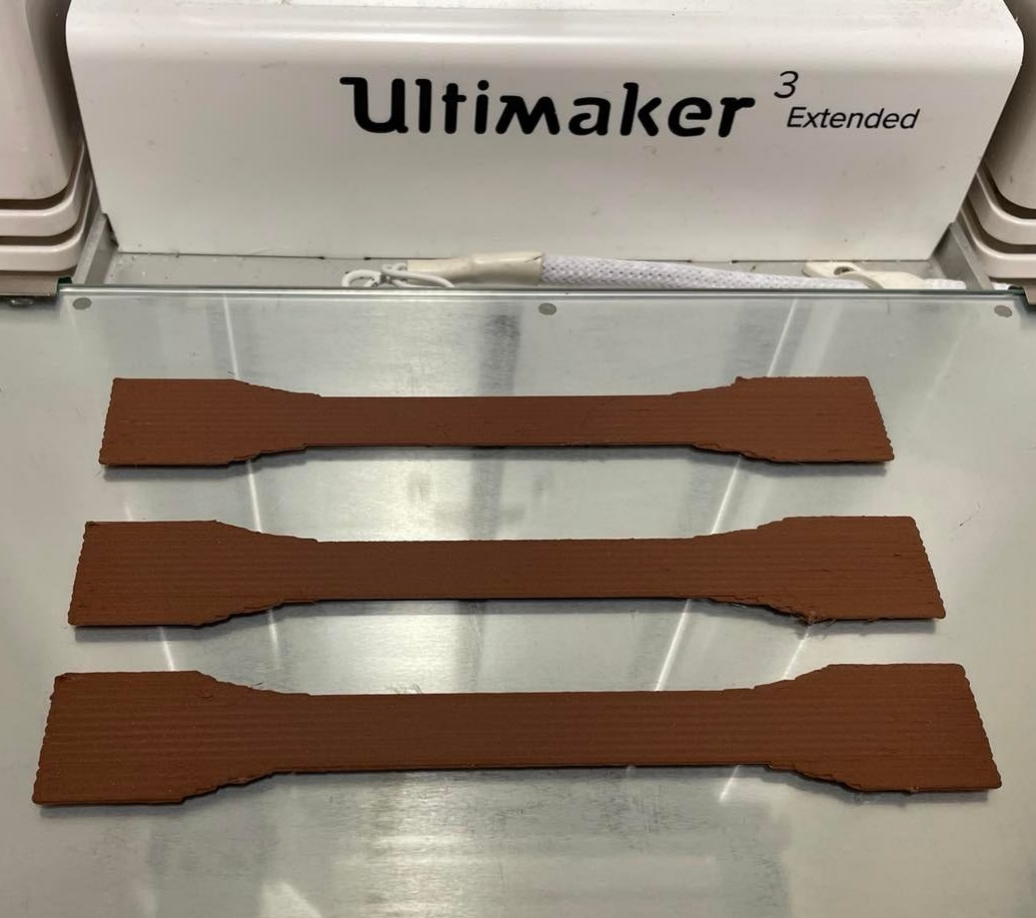
FDM equipment and the printed specimens.

All layers of specimens are FDM printed unidirectionally with 0° layer orientation. The material properties from the manufacturer are given in Table 2 for copper-reinforced PLA composites. We claim that the manufacturer parameters were measured by using a mold specimen such that the porosity was expected to be almost zero. Therefore, these values are assumed as an upper limit for FDM copper-reinforced PLA. We provide a photo of used FDM equipment after printing in Figure 1.

**Figure 2:**
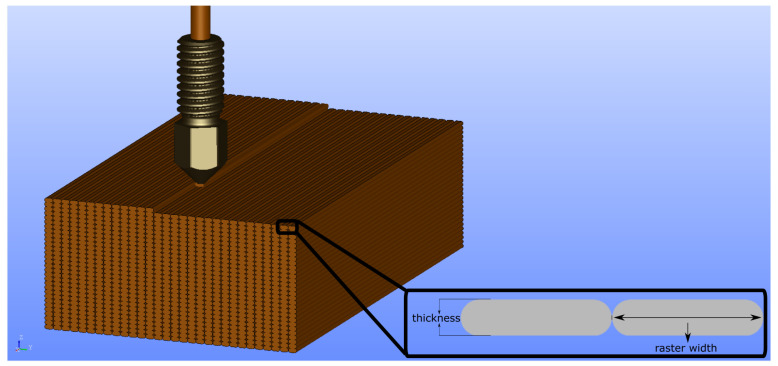
Representation of the raster width and layer thickness parameters.

**Figure 3:**
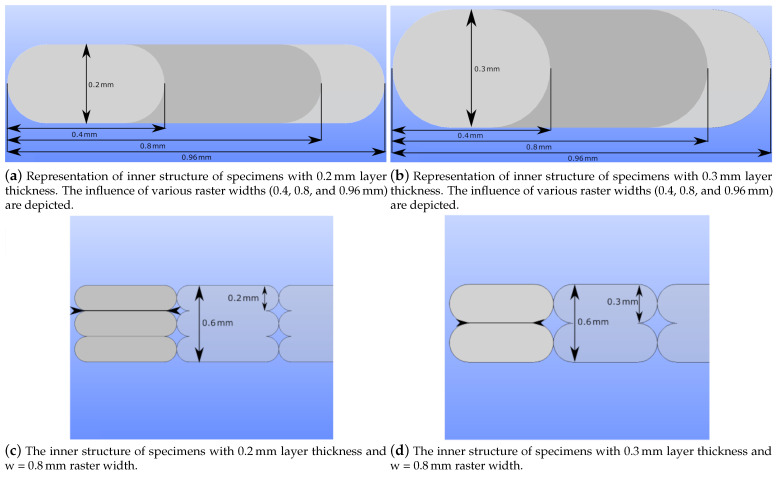
An illustration of how raster width and layer thickness variations affect the inner structure in FDM-printed polymers. Black arrows demonstrate the interfiber (interface between fibers) regions in (**c**,**d**). The change in fiber volume with different raster width settings is represented in (**a**,**b**). The symbols h and w represent layer thickness and raster width of the specimens, respectively.

Fused deposition modeling is a layer-by-layer production method. Thus, the layer thickness is one of the critical parameters which can vary the product’s mechanical properties. A relatively thicker layer causes lower dimensional accuracy; however, it hastens the production. On the other hand, a thinner layer increases the strength [33,45]. We emphasize that “fiber” is used as jargon for denoting the deposited materials onto the build plate (see detail view of Figure 2 for the fibers and the process parameters that affect the fiber geometries). Only copper particles are reinforced PLA matrix in this study, and no other fiber reinforcement is studied herein. Raster width is a key FDM process parameter that affects the fiber geometry, (see Figure 2 and Figure 3a,b). A relatively broader raster width increases the fiber diameters, which reduces the number of fibers in a layer and hence less porosity in the structure (see Figure 3a,b). Moreover, it influences the contact surface between adjacent layers.

Porosity is expressed here as the ratio of voids over the total area. The infill ratio as a process parameter represents the total amount of deposited materials to the whole geometry. An infill ratio lower than 100% means voids between deposited materials, and substructures in the product, which forms a metamaterial [44,46]. In this work, all specimens were produced with 100% infill ratio, and the metamaterials were not examined. Nevertheless, the porosity is not zero, due to process technology employed in FDM, even for 100% infill ratio [33]. Process parameters (see Figure 2 for the parameters, which are studied herein) change the porosity in additive manufacturing, leading to different inner structures.

In this study, we demonstrate how layer thickness and raster width modify the inner structure, thereby the mechanical properties. For investigating this phenomenon, specifically, we have examined FDM-printed specimens with two different layer thickness values (0.2, 0.3 mm) and three different raster widths (0.4, 0.8, 0.96 mm). We present the experimental design and number of specimens in Table 3.

Change in thickness alters the contact areas of neighboring layers. We provide Figure 3 for visualizing this phenomenon. When we increase the raster width, the adjacent layers obtain larger contact areas. More contact areas may lead to more molecular diffusion during FDM printing, stronger bond formation, and less porosity at the macroscale.

**Figure 4:**
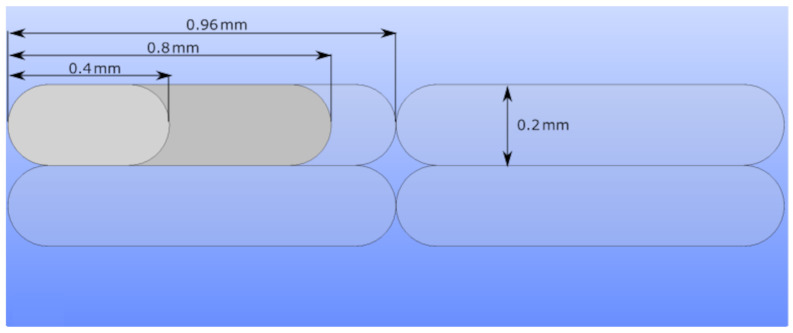
Representation of the fibers with different raster widths on the FDM mesostructure with 0.2 mm layer thickness.

In Figure 3c,d, we demonstrate the difference between the contact areas of two layers. Changes in layer thickness result in different inner structures and mechanical properties, specifically when different slicer software is employed. Slicers have significant effects on flow characteristics [47], which influence the shapes of deposited fibers in FDM. In this study, we used an Ultimaker Cura slicer. Deposited fibers become more elliptical with decreasing layer thickness, which leads to relatively higher contacts between adjacent layers and stronger bonding (see Figure 3d). This situation also results in lower porosity on the macroscale. Change in layer thickness and raster width parameters are depicted together in Figure 4 for a better illustration.

### 2.2. Tensile Tests

**Figure 5:**
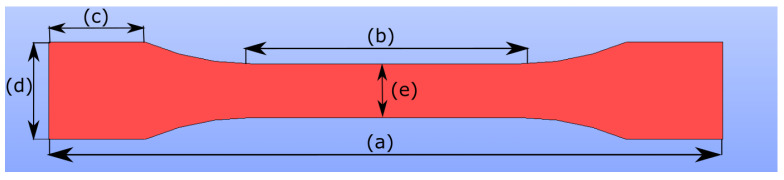
Tensile test specimen geometry is designed according to ISO 527-2. The specifications are given in Table 4. The symbols, a, b, c, d, e denote overall length, gauge length, length of grip section, width at ends, width at narrow portion, respectively.

**Figure 6:**
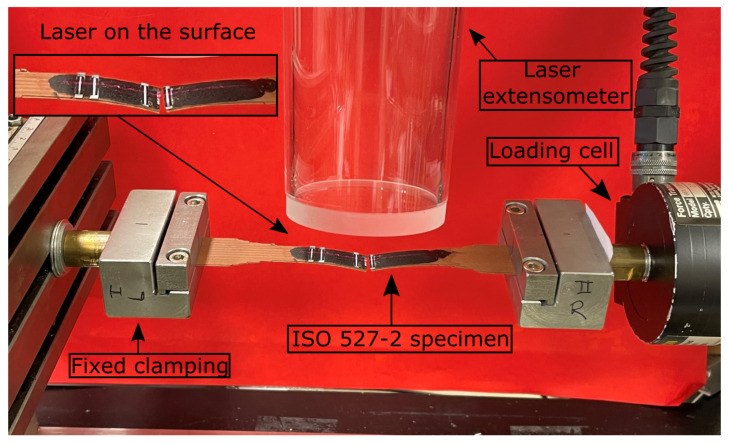
A clamped ISO 527-2 specimen into a MTS Tytron 250 testing equipment with a laser extensometer (**top**), a loading cell (**right**), and a fixed mounting side (**left**). Displacement is applied horizontally through the right mounting side. Laser is visible on the surface of the specimen (**top**–**left**).

Displacement-controlled uniaxial tensile tests are performed on the specimens, which are prepared according to ISO 527-2 standards. Specimen specifications are given in Table 4 and in Figure 5. The experimental setup and front view of a clamped specimen into the test machine are illustrated in Figure 6. For more information about the proper geometry selection and process optimization for FDM printing, we refer to [10].

MTS Tytron 250 (MTS Systems Corporation, Eden Prairie, USA) test equipment is used for tensile testing. Loading cell (N=±250 N with an accuracy of 0.2 percent) is controlled and monitored by the device’s own software (Stationsmanager V 3.14). Experiments were performed horizontally with a 2 mm/min speed through the right side (see right of Figure 6).

**Figure 7:**
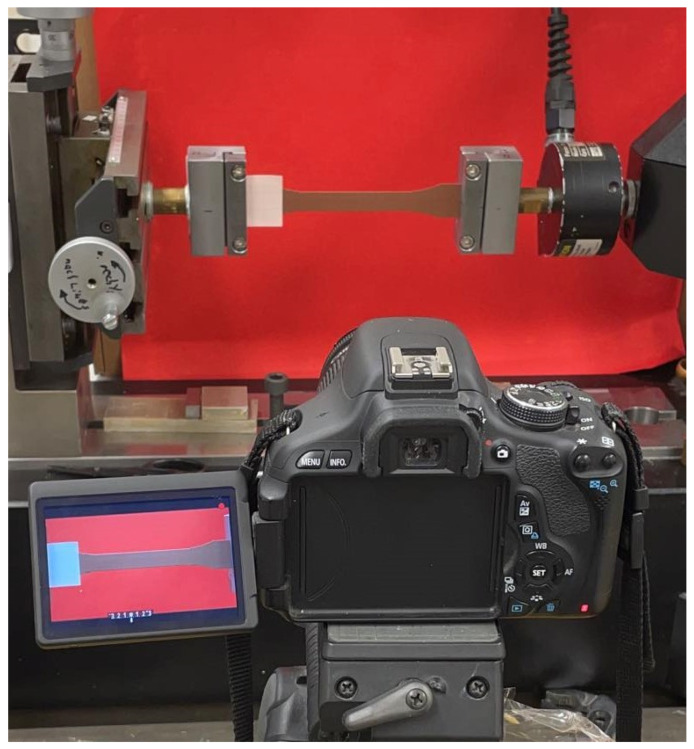
Canon EOS 600D camera for measuring deformations on the specimen surfaces.

Nearly frictionless displacement is achieved due to hydraulic air-film-bearing system. A laser extensometer is conducted for measuring strain (Figure 6). Furthermore, deformations on the surfaces are observed (see Figure 7). Canon EOS 600D camera (4272×2848 pixels, 1 picture/2 s) is used, and the displacements on the deformed surfaces from recorded pictures are visualized. Engineering stress and strain were computed from experimental force and displacement measurements. Postprocessing was carried out by the device-own software for calculating ultimate tensile strength (UTS) and Young’s modulus. Five samples from each group (see Table 3) were tested for reliable results.

### 2.3. Optical Microscopy and Porosity Analysis

**Figure 8:**
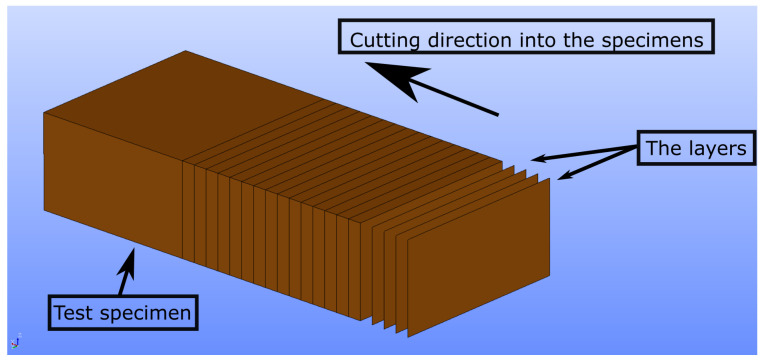
Representation of layer cutting from the surface by rotary microtome, CUT 4055 (Micro Tec Laborgeräte GmbH, Walldorf, Germany). The surfaces and also deeper parts of the specimens were analyzed through this method.

**Figure 9:**
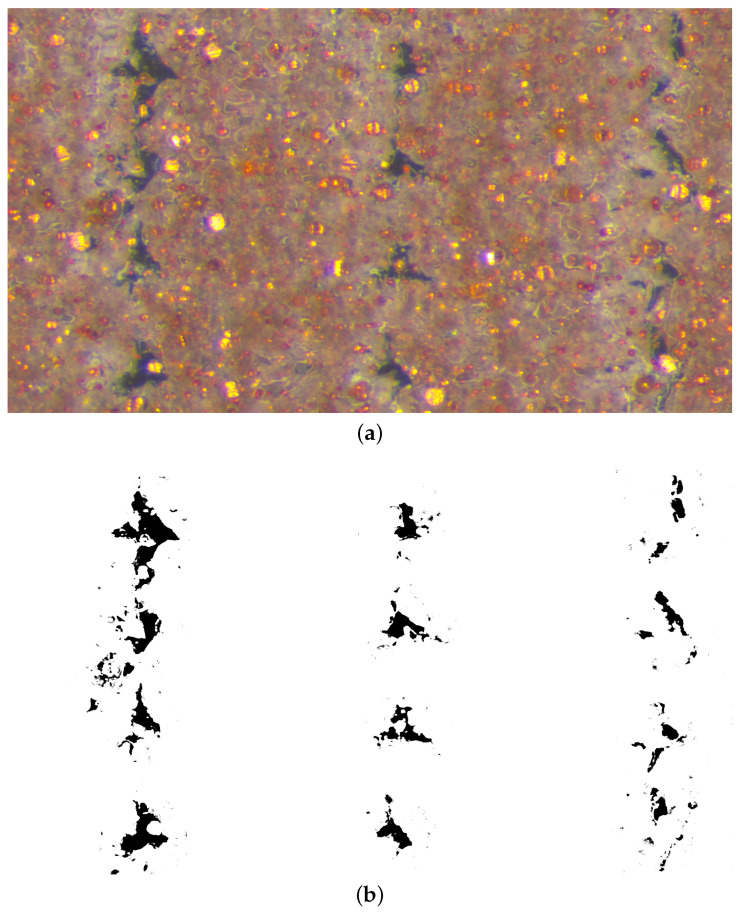
An example binarization and porosity analysis by digital image correlation code. (**a**) Microscopy image of the specimen with 0.3 mm layer thickness and 0.8 mm raster width. (**b**) Binarized image of (**a**).

Leica Wild M3C Heerburg-type polarized microscope was used for microscopy analysis. We examine how various process parameters affect the inner structures. One sample of each configuration (see Table 3) was cut in the middle. Manual cutting was preferred rather than milling because heat generation may alter the phases of the inner structure. Then, thin layers from the surface were sliced by using a rotary microtome, namely CUT 4055 (Micro Tec Laborgeräte GmbH, Walldorf, Germany). We illustrate in Figure 8 how we sliced the layers from the surfaces and reached the specimens’ inner parts. The layers from different positions (see Figure 8 for layer positions) were examined by means of microscope, and the images were saved as .png files. Totally, 182 microscopy images were taken. We provide an example analysis in Figure 9.

After microscopy, a digital image correlation (DIC) code was developed and used for calculating porosity from 2D images. DIC code was written in Python language with Tensor-flow packages [48]. The code first imports the microscope images using Opencv (cv2) [49], and they are transformed into 2D NumPy arrays [50]. Then, they are binarized by applying color range thresholds to the NumPy arrays, and all colors in images are reduced to black and white. Binarization helps to calculate porosity effectively and prevents possible errors in further steps. After that, the code employs a machine learning algorithm, namely K-means cluster. This algorithm groups the data with similar characteristics. At last, the porosity is calculated by counting the pixels, which are collected from the K-means cluster. For more information about DIC code development, binarization, and employment of machine learning algorithms in DIC code, we refer to [33].

### 2.4. Scanning Electron Microscopy (SEM) and Energy Dispersive X-ray Spectroscopy (EDS)

**Figure 10:**
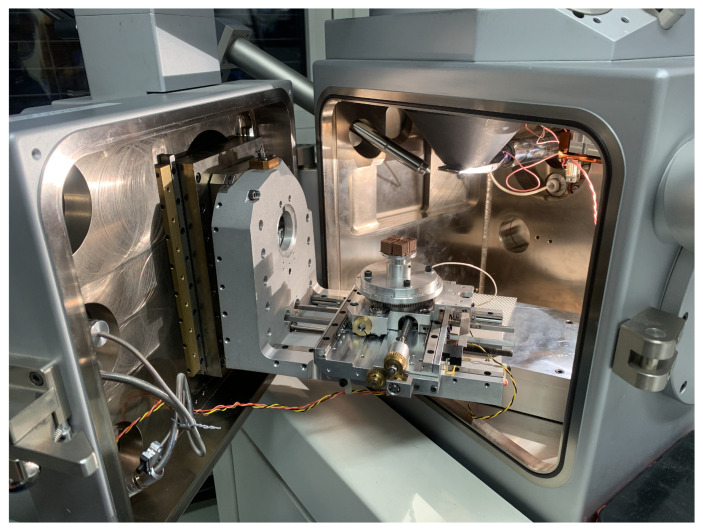
CamScan MaXim 2040-type SEM equipment was employed for microscopy analysis.

Scanning electron microscopy (SEM) was carried out using a CamScan MaXim 2040 microscope (CamScan Electron Optics Ltd, Cambridge, UK), and energy dispersive X-ray spectroscopy (EDS) was performed by Bruker XFlash 6 30 detector (Bruker Nano, Berlin, Germany). The acceleration voltage was 30 kV. The EDS analysis was utilized with ESPRIT 2.0 software (Bruker Nano, Berlin, Germany). We provide a photo of the used SEM equipment and the specimens during preparation in Figure 10.

## 3. Results and Discussion

### 3.1. Mechanical Characterizations

The tensile tests were performed to analyze how different process parameters affect the mechanical properties. We understood that the elasticity modulus was increased when the layer thickness was decreased (see Table 5). A lower thickness results in a higher number of layers and, hence, larger contact lines between adjacent fibers, as we schematically describe in Figure 3c,d. Greater contacts enable higher molecular diffusion during FDM printing from deposited molten polymers to already solidifying ones. This leads to a stronger bond formation and thus a higher elasticity modulus.

We also stress that the altered contacts due to a lower layer thickness decreased the porosity ratios. In this study, similar characteristics of FDM-printed polymers regarding the layer thickness–porosity relationship for FDM polymer composites were observed. A lower porosity resulted in denser specimens and hence a higher elasticity modulus.

A larger raster width increases the elasticity modulus analogous to a lower layer thickness. We schematically describe how the inner structure is affected by different raster widths in Figure 3a,b. A larger raster width leads to greater contact lines with neighboring fibers. It increases the amount of printed materials and results in a lower porosity. This situation is depicted in Figure 4.

We emphasized that both a lower layer thickness and a larger raster width increased the elasticity modulus analogously. However, these parameters altered the inner structures differently (see Figure 3). As a result, if a higher stiffness is intended, the layer thickness is to be lowered, and the raster width needs to be increased. We also stress that all these changes in the process parameters depend on the flow characteristics of the printed materials, slicer/printer types, and environmental conditions. Higher UTS results are measured with a lower layer thickness and larger raster widths. We provide Table 5 for the experimental UTS results. The reason is related to the aforementioned stronger bond formation between the adjacent layers and fibers due to greater contacts and lower porosities.

**Figure 11:**
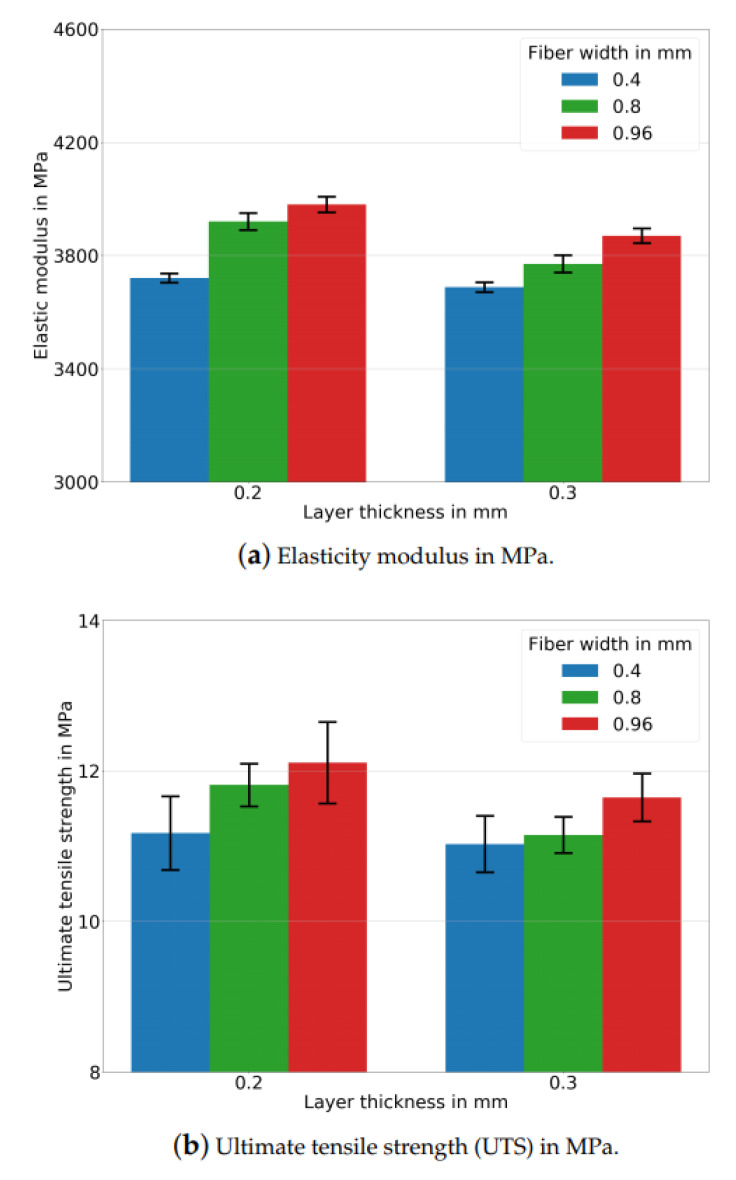
Comparison of different layer thickness values using three different raster widths of 0.4, 0.8, and 0.96 mm. Arithmetic mean results of many tests with error bars are given.

The engineering stress results are calculated by dividing the measured force to the cross-section area of the specimen, and the engineering strain is computed by dividing the measured elongation from the extensometer to the initial length of the specimens. Admissible relative errors (less than 5%) are obtained for the elasticity modulus and ultimate tensile strength results. Two specimens from each group are tested with a laser extensometer, the other ones are measured by fitting their machine compliance with a fifth-degree polynomial fit, and they are analyzed by means of a high-resolution camera. We provide Figure 11 for the test results with error bars of all the experimental groups. The error calculations are carried out as follows,

(1) $$R=\frac{S}{m}\times 100,$$

where *R*, *S*, and *m* denote the relative standard error as percentage, standard deviation, and the arithmetic mean of all samples, respectively.

**Figure 12:**
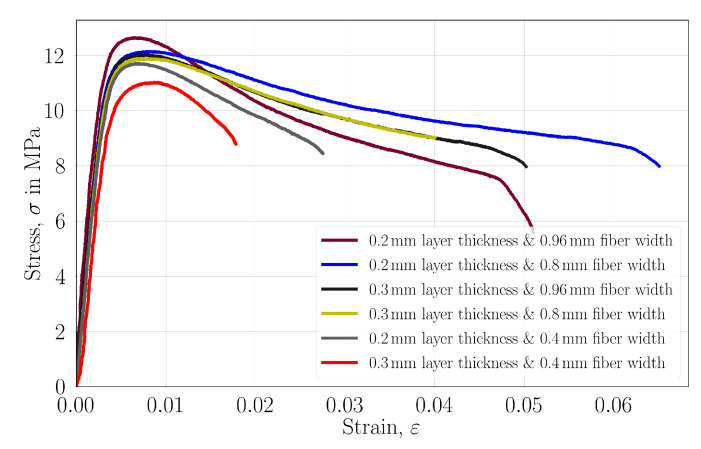
Engineering stress vs. engineering strain for specimens with different raster widths and layer thicknesses.

The stress–strain curves are provided in Figure 12 for each experimental configuration. Generally, longer softening behavior is observed in the specimens with lower porosities. They reached an ultimate tensile strength in the higher strains and have a mostly higher strain at the failures. Here, the energy release is meant to be the area under the curves. We interpret that altering the inner structure is important to increase the mechanical properties; however, strong bond formations between fibers and layers are needed for long softening behavior as it also mostly happened in the specimens with lower porosities. FDM-printed polymers generally show lower mechanical properties than mold specimens when they have weak bonding between adjacent layers and fibers [51]. This situation is proven here, as the specimens with higher porosities exhibit lower mechanical properties and energy release.

**Figure 13:**
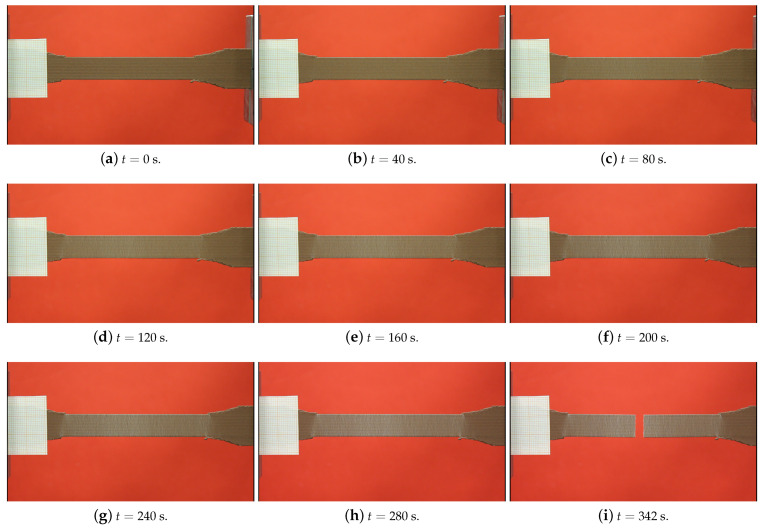
High-resolution Canon EOS 600D camera (4272×2848 pixels, 1 picture/2 s) was used for recording the tensile tests, and we provide here an example record by dividing the video to 40 s.

**Figure 14:**
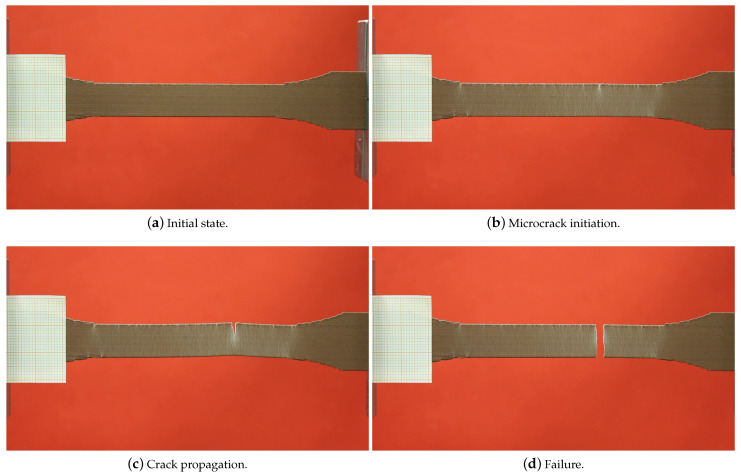
Different stages of the failure. The initiation of microcrack and its propagation occurred in 2 s. After Figure 13c, the specimen failed immediately.

We observed the deformation behavior of some specimens during the tensile testing with a high-resolution camera. The camera record of a specimen is provided in Figure 13. The test took 5 min and 45 s. We divided the camera record into every 40 s in order to show the elongation and the failure. The deformation became visible after t=120 s, by the white color on the specimen (see Figure 13d). A small microcrack is nearly visible in t=280 s (see Figure 13h, in the center of the specimen). This propagated in the following seconds up to t=341 s and led to specimen failure in t=342 s. (Figure 13i). A brittle failure is observed. We stress that there was a homogeneous stress distribution and hence deformations between the shoulders of the specimens, which was understood by the homogeneous distribution of the white colors in Figure 13. An adequate failure position was seen as the rupture occurred in the center of the specimen. This is a proof of optimum FDM printing and testing conditions. For more information, we refer to [10]. Moreover, another camera record during the tensile testing is provided in Figure 14, as the microcrack initiation and propagation stages are relatively more apparent.

### 3.2. Optical Characterization

**Figure 15:**
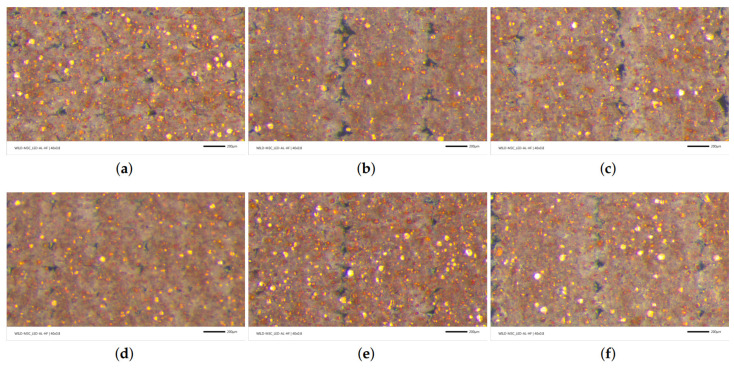
Microstructure images of FDM-printed specimens with different process parameters. (**a**) 0.3 mm layer thickness and 0.4 mm raster width. (**b**) 0.3 mm layer thickness and 0.8 mm raster width. (**c**) 0.3 mm layer thickness and 0.96 mm raster width. (**d**) 0.2 mm layer thickness and 0.4 mm raster width. (**e**) 0.2 mm layer thickness and 0.8 mm raster width. (**f**) 0.2 mm layer thickness and 0.96 mm raster width.

We have investigated the effect of different layer thicknesses and raster widths by microscopy analysis. The microscope images are depicted in Figure 15. We observe that decreasing the layer thickness increases the number of porous areas in the images because FDM prints more layers and the amount of material in a cross-section. The fibers become relatively more elliptical, and consequently, the void areas become smaller. This results in a lower porosity for FDM polymer composites, as we also found out for FDM polymers in [33]. A larger raster width does not change the geometry of porous areas; however, it decreases the number of them and lowers the porosity. In addition to visual observations, we calculated the porosity from each image by the aforementioned image code with machine learning. The results are provided in Table 6. A DIC porosity analysis justifies the interpretations from the visual inspections. The porosity values vary from 1 up to 5.6%. We emphasize that the inner structure and the porosity depended heavily on the process parameters, namely the layer thickness and raster width. As a result, we interpret that a larger raster width and lower layer thickness decrease the porosity.

**Figure 16:**
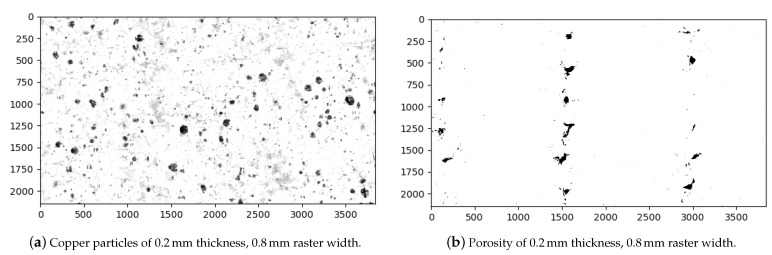
Binarized micrographs with two different modes ((**a**) representation of particles and (**b**) microporous areas) for the specimens with 0.2 mm layer thickness and 0.8 mm raster width configuration. The original microscope image of this configuration can be found in Figure 15e.

In Figure 15, the dark gray-black areas denote the micropores. Brown is understood as the PLA or copper-reinforced PLA. Yellow and somewhat yellow/brown shiny particles are interpreted as copper particles. The gray-black levels of the images represent the measure of porosity in the structure. Because the yellow/brown shiny colors are located everywhere in the microscope images (see Figure 15), and their intensities are similar in each of the experimental configurations (see Figure 15 and Figure 16a), we interpret that the copper particles are homogeneously distributed.

In the 0.4 mm raster width configurations (see Figure 15a–d), there are relatively smaller contact areas between the neighboring fibers. This leads to lower molecular diffusion and hence brittle behavior, which is well confirmed by Figure 12. It is visible that the specimen with 0.3 mm has a higher porosity than the 0.2 mm ones, which is also measured by the image code (see Table 6).

In the 0.8 mm and analogously in the 0.96 mm raster width configurations (see Figure 15b–f), there are relatively larger contact lines between the fibers. Therefore, we assume that the higher molecular diffusion occurred, especially at 0.96 mm. As seen in Table 6, the porosity decreased to 1% by optimizing the parameters. Additionally, we interpret that enough molecular diffusion leads to interlayers as good as the fiber itself in 0.96 mm, because we do not observe any significant color difference between the aforementioned areas in Figure 15c–f. We provide Figure 16 for representing the binarization of the copper particles and microporous areas, separately, using the digital image analysis with machine learning.

**Figure 17:**
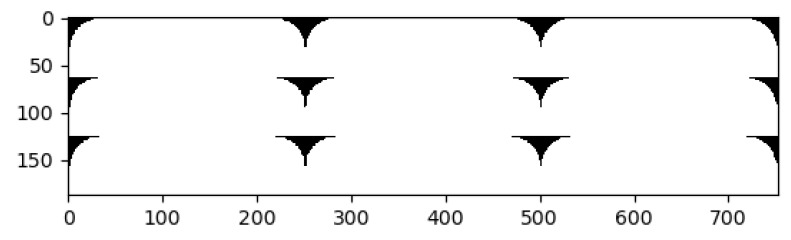
Binarized CAD image of the specimen with 0.2 mm layer thickness and 0.8 mm raster width.

A binarized CAD image of 0.2 mm layer thickness−0.8 mm raster width is depicted in Figure 17. We observe that the microporous areas and the connections between the fibers are distributed more uniformly in the CAD images. Due to the process conditions of FDM, some lacking contacts in the structure may occur. This may lead to lower molecular diffusion and hence a decrease in the properties.

**Figure 18:**
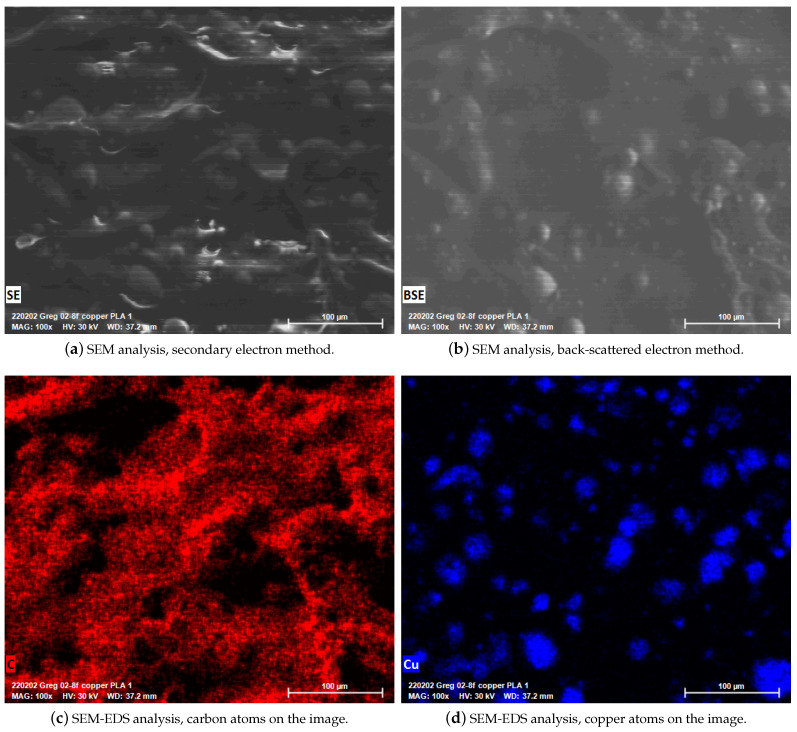
SEM-EDS analysis results. Different imaging mods such as SE and BSE are employed. By means of EDS analysis, different materials on the same topology are depicted in (**c**,**d**), respectively. All SEM-EDS images were taken at 100× magnifications.

We provide SEM images and depict the EDS analysis results in Figure 18. Two different imaging modes are utilized as the secondary electron (SE) (see Figure 18a) and back-scattered electron (BSE) methods (see Figure 18b). Back-scattered electrons are reflected back from the samples, and most electrons do not lose their energy on the specimen surface. Therefore, it is assumed that BSEs make elastic interactions with specimens. Secondary electrons originate from the surface of FDM-printed copper-reinforced PLA specimens. Hence, it is interpreted that SEs are derived by inelastic interactions.

Secondary and back-scattered electrons provide different types of information due to their aforementioned generation and interaction differences. Secondary electron images provide more detailed topological features because they originated from the surface, as seen in Figure 18a. Back-scattered electron images provide high accuracy for differentiating the materials from different atomic numbers. The materials seem brighter if they have higher atomic numbers in the image [52]. For instance, in Figure 18b, the copper particles seem more apparent than the PLA matrix as the atomic number for copper is 29, whereas carbon’s is 6.

An EDS analysis allows to differentiate the various substances, such as carbon and copper, from the same topology and depict them in different images. Due to the high magnification of the SEM and the substance analysis feature of the EDS, we analyze the copper particles separately (see Figure 18d).

## 4. Multiscale Homogenization

### 4.1. CAD Preparation

All the preprocessing steps, i.e., CAD generation, marking boundary conditions, and triangulation (mesh generation), are accomplished in Salome 9.3.

For the microscale simulations, two representative volume elements (RVEs) are prepared, which consist of spherical copper particles and a PLA matrix. The particle size distribution is obtained from the filament manufacturer company. In the RVE, 0.46 μm diameter particles are generated as it is nearly the arithmetic mean of the size distribution. For the microscale simulations, the volume percentage % of the copper particles is necessary. The calculation of the vol. percentage % method is implemented from [53].

**Figure 19:**
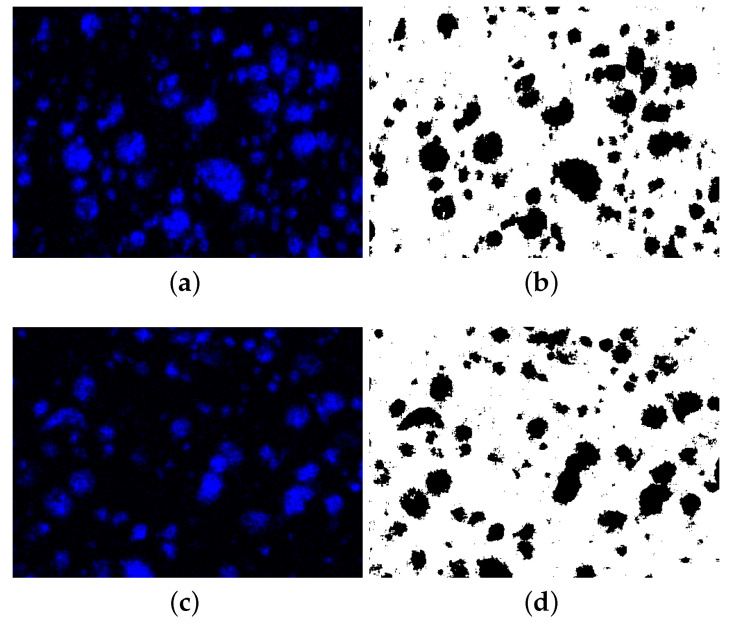
Original and binarized SEM-EDS images for calculating volume percentage of copper particles in the filaments. SEM-EDS of copper particles for the specimens with 0.2 mm layer thickness–0.4 mm raster width and 0.2 mm layer thickness–0.8 mm raster width are depicted in (**a**,**c**), respectively. Their binarized images are provided in (**b**,**d**), respectively.

According to this method [53], the SEM-EDS analyses are carried out because they enable the differentiation of the various substances in the SEM images. In this work, the EDS images of the copper particles from two different experimental configurations, namely 0.2 mm layer thickness–0.4 mm raster width and 0.2 mm layer thickness–0.8 mm raster width, were examined (see Figure 19). In order to distinguish the copper particles clearly, the SEM-EDS images were binarized (see Figure 19, right). After the binarization, the copper particles are represented by black colors while the rest are illustrated by white. Ultimately, the relative percentage of black and white is computed, which allows for the vol. percentage % to be calculated. The binarization and computations were carried out by means of our digital image correlation code with its machine learning algorithm (see Section 4.2, optical microscopy and porosity analysis). For more information about the digital image correlation code preparation and its structure, we refer to [33].

**Figure 20:**
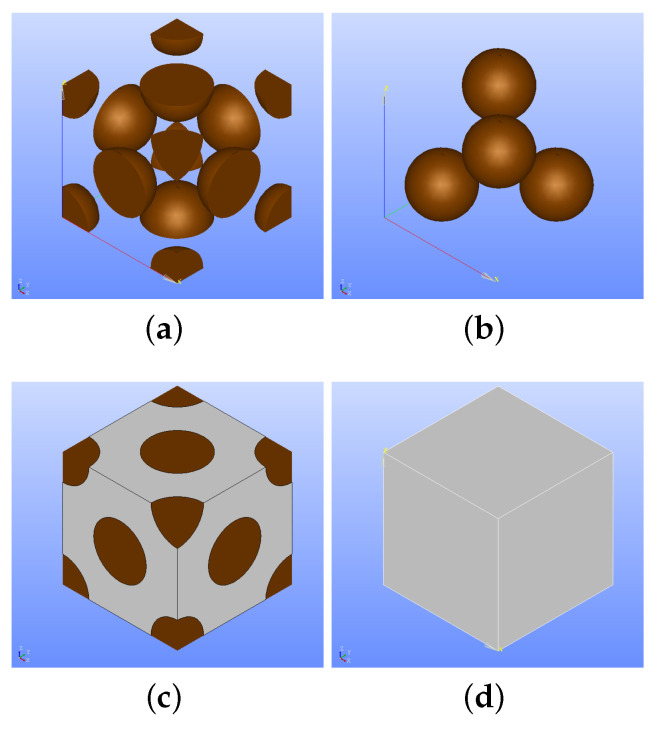
Illustrations of two different representative volume elements (RVEs) with different particle orientation. (**a**) Fillers are on the surface of RVE, $V_{f}$ = 20.4%. (**b**) Fillers are embedded in the matrix of RVE, $V_{f}$ = 20.4%. (**c**) Outer view of (**a**), RVE with the particles on the surface. (**d**) Outer view of (**b**), particles are embedded in the PLA matrix.

After the aforementioned analysis, we find out that the volume percentage of the copper particles is 20.4% as a mean value. Two RVEs are generated with different positions of particles. The particles are homogeneously distributed on the surfaces of the volume in RVE1 (see Figure 20a,c), and they are embedded in the matrix without any surface connection in RVE2 (see Figure 20b,d). We provide the RVE images in Figure 20.

**Figure 21:**
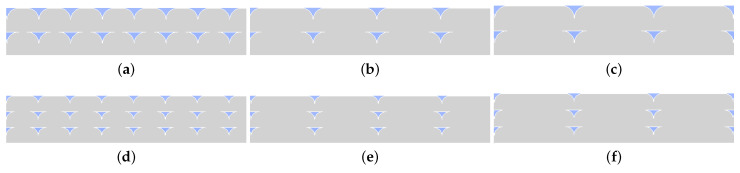
Cross-section microstructure of an ideal configuration constructed in CAD. (**a**) 0.3 mm layer thickness and 0.4 mm fiber width; (**b**) 0.3 mm layer thickness and 0.8 mm fiber width; (**c**) 0.3 mm layer thickness and 0.96 mm fiber width; (**d**) 0.2 mm layer thickness and 0.4 mm fiber width; (**e**) 0.2 mm layer thickness and 0.8 mm fiber width; (**f**) 0.2 mm layer thickness and 0.96 mm fiber width.

For the mesoscale simulations, six CAD geometry are prepared for each experimental configuration (see Table 3), regarding their layer thickness and raster width variations. Each configuration has been analyzed by optical microscopy and then by our digital image correlation code with its machine learning algorithm. By means of the analysis, the mesostructural properties of different configurations, such as the contact lines between the adjacent fibers and layers, the contact geometry, and the final fiber shape after solidification, are obtained. By considering these features, the CAD geometries are generated, representing ideal cases. We depict the cross-section of the CAD models in Figure 21.

**Figure 22:**
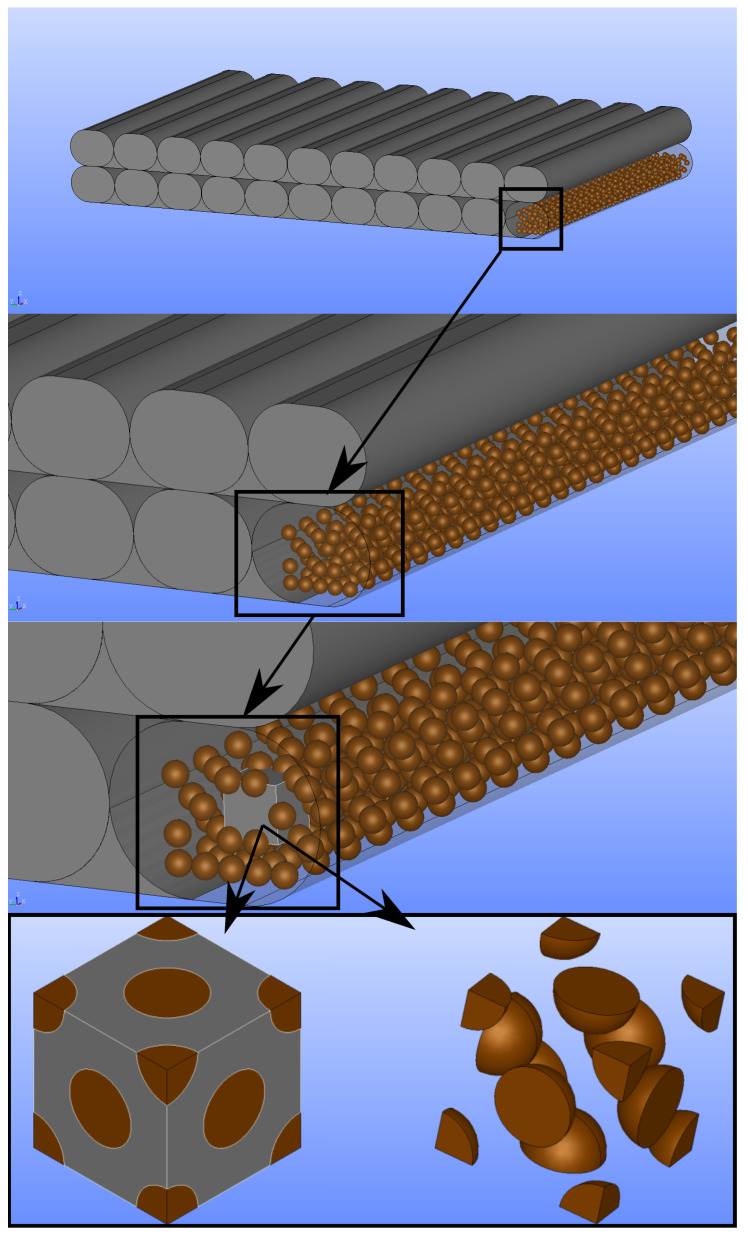
Mesostructure and the copper particles in CAD image of 0.3 mm thickness, 0.4 mm raster width. We illustrate how we provide multiscale simulations step by step for aforementioned configuration and RVE1 (particle contacts with surface).

Due to the process conditions, solidification conditions, and temperature effects in the laboratory experiments, the porosities are lower than their ideal cases. Therefore, the porosities of the CAD images are assumed as the upper thresholds for manufacturing. The values of the CAD images are presented in Table 7, and Table 6 shows the porosities measured by the manufactured samples. We provide a schematic representation of the copper particles in the layered mesostructure for the 0.3 mm layer thickness−0.4 mm raster width specimen and a generation of the representative volume element for its microscale analyses in Figure 22.

### 4.2. Microscale Simulations

Asymptotic homogenization is utilized with periodic boundary conditions for determining the homogenized material stiffness matrix on the microscale, which consists of copper particles and a PLA matrix. We model all these ingredients as linear elastic isotropic materials. The system at the microscale is assumed to be heterogeneous, and we compute the homogenized effective stiffness matrix. The homogenization method is implemented from [44,54,55]. We employ the standard procedure of the finite element method [56] by solving the weak form:

(2) $$\int_{\Omega_{p}}C_{ijkl}^{m}L_{abkl}\frac{\partial \delta \varphi_{abi}}{\partial y_{j}}=0,$$

where “m” denotes the microscopic amounts. $C_{ijkl}^{m}$ is the stiffness matrix of the materials in the microscale and $L_{abkl}=\delta_{ak}\delta_{bl}+\frac{\partial \varphi_{abk}}{\partial y_{l}}$. φ is unknown tensor and needs to be numerically solved in order to find homogenized stiffness properties, which is carried out by means of the FEniCS project [57,58]. The Galerkin procedure is utilized because we use finite-dimensional Hilbetian Sobolev space for trial functions and the same space for test functions as well.

**Figure 23:**
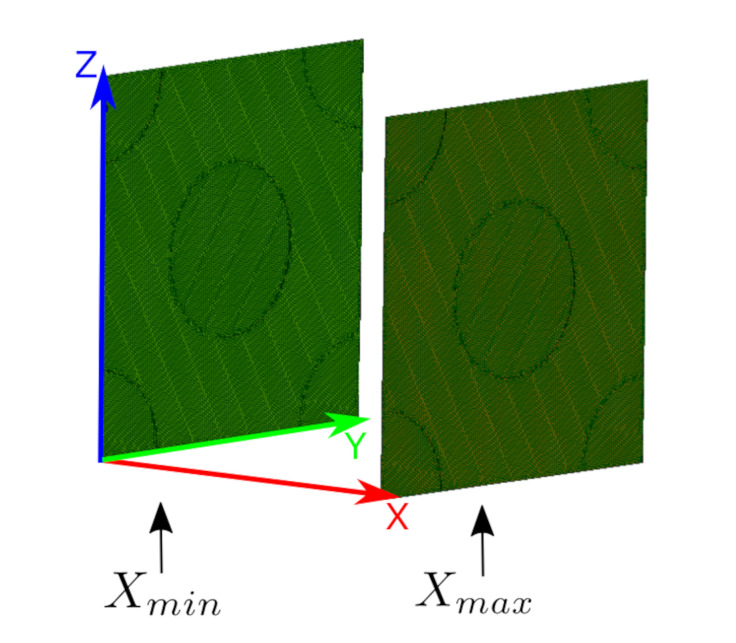
Representation of the surface mesh of RVE1. The same mesh is generated on the corresponding surfaces along *X* axis, namely $X_{max}$ and $X_{min}$.

We use periodic boundaries, which means that the degrees of freedom on each node of a surface need to be equivalent to the corresponding nodes on the opposite surfaces. More specifically, $X_{max}$ and $X_{min}$ (see in Figure 23) are corresponding surfaces along the *X* axis, and the same meshes are generated on them. All nodes on these surfaces have the same *Y* and *Z* coordinates. All the boundaries are Dirichlet type. In order to provide the aforementioned feature of the corresponding surfaces, we generate mesh in Salome 9.3 by applying the NetGen and Mephisto algorithms.

After solving the aforementioned weak form, we apply these results in the following equation, and finally, the homogenized effective stiffness matrix, $C_{abcd}^{M}$, is determined as follows

(3) $$C_{abcd}^{M}={\bar{C}}_{abcd}=\frac{1}{V}\int_{\Omega_{p}}C_{ijkl}^{m}L_{abij}L_{cdkl}\;dV.$$

We model both copper particles and PLA as a linear elastic isotropic material in the microscale. The elasticity modulus of copper particles is 100 GPa, which is about the standard value for pure copper, and their Poisson’s ratio is 0.34. PLA has a 3 GPa elasticity modulus, which is provided by the filament manufacturer, and it has 0.35 Poisson’s ratio. Moreover, we assume perfect bonding between particles and matrix phases in these simulations. By means of the aforementioned asymptotic homogenization, we compute and provide the stiffness matrix for RVE1 (see Figure 20a,c), whose particles are aligned on its surface as follows

(4) $$C=\left[\begin{array}{cccccc} 6700.0 & 3397.4 & 3397.9 & 0 & 0 & 0\\ 3397.4 & 6699.6 & 3397.8 & 0 & 0 & 0\\ 3397.9 & 3397.8 & 6700.3 & 0 & 0 & 0\\ 0 & 0 & 0 & 1725.4 & 0 & 0\\ 0 & 0 & 0 & 0 & 1725.3 & 0\\ 0 & 0 & 0 & 0 & 0 & 1725.7 \end{array}\right],$$

as well as for RVE2 (see Figure 20b,d), whose particles are embedded in its matrix, reads

(5) $$C=\left[\begin{array}{cccccc} 6699.1 & 3397.6 & 3397.7 & 0 & 0 & 0\\ 3397.6 & 6700.1 & 3398.0 & 0 & 0 & 0\\ 3397.7 & 3398.0 & 6700.3 & 0 & 0 & 0\\ 0 & 0 & 0 & 1724.5 & 0 & 0\\ 0 & 0 & 0 & 0 & 1724.3 & 0\\ 0 & 0 & 0 & 0 & 0 & 1724.6 \end{array}\right].$$

Because the elastic parameters, $C_{1111}=C_{2222}=C_{3333}$, $C_{1122}=C_{1133}=C_{2233}$ and $C_{2323}=C_{3131}=C_{1212}$ are almost equal in Equation (4) as well as in Equation (5), we understand the homogenized materials of RVE1 and RVE2 are isotropic. Therefore, we only calculate $E_{1}$ from the aforementioned equations as it is comparable with the mechanical data provided by the filament company (Table 2). The comparison is given in Table 8. We refer to [59] for more details about determining the engineering constants from the stiffness matrix.

We observe that the elasticity moduli, $E_{1}$, of RVE1 and RVE2 are almost equal, and there is no significant variation. There is an admissible difference (4.6%) between the elasticity moduli of the filament and the RVEs. Hence, we interpret that the microscale simulations are validated, and they are used as the input data in the multiscale simulations, which is explained in the following.

### 4.3. Mesoscale Simulations

Computational homogenization is employed for the mesoscale simulations. The computations are carried out by the finite element method (FEM) for the space discretization. Suitable mesh is generated after an *h*-convergence analysis. We refer to [56] for more details about the FEM implementation and linear elasticity. FEM software has been prepared in the Python language with the open-source packages of the FEniCS project [57,58]. ParaView 5.6 is used for postprocessing. The copper-reinforced PLA is modeled as a linear elastic material with the stress–strain relation of Hooke’s law as follows:

(6) $$\begin{array}{c} \sigma_{ij}=C_{ijkl}\varepsilon_{kl}. \end{array}$$

The mesoscale simulations are uniaxial tensile tests, where the Dirichlet boundary conditions are applied for clamping one end, and the other is moved by a given displacement along the tensile axis. The deformation is measured by linear shape functions via standard Lagrange elements with the Galerkin procedure. The gravitational forces have been neglected as the displacement is caused mainly by surface loading. We refer to [33,46] for more details about the computational homogenization of FDM polymers and FDM polymer-metamaterials.

A detailed microscale model allows the calculation of the material properties at the mesoscale accurately. In this section, we model the microscale of copper-reinforced PLA as an isotropic material model with an elasticity modulus of 4210 MPa (see Table 1) and Poisson’s ratio: 0.35.

The transverse isotropic material model (symmetry axis: $x_{2}$ and $x_{3}$ directions) has been employed for modeling the specimens on the mesoscale because all the specimens are FDM printed unidirectionally, which means that each layer has the same layer orientation. Its compliance matrix in Voigt’s notation is given as follows

(7) $$S=\left[\begin{array}{cccccc} \frac{1}{E_{1}} & -\frac{\nu_{12}}{E_{1}} & -\frac{\nu_{12}}{E_{1}} & 0 & 0 & 0\\ & \frac{1}{E_{2}} & -\frac{\nu_{23}}{E_{2}} & 0 & 0 & 0\\ & & \frac{1}{E_{2}} & 0 & 0 & 0\\ & & & \frac{2(1+\nu_{23})}{E_{2}} & 0 & 0\\ & \mathrm{sym} & & & \frac{1}{G_{12}} & 0\\ & & & & & \frac{1}{G_{12}} \end{array}\right]\;.$$

Six engineering parameters are required to be characterized for this material model, namely: elasticity moduli, $E_{1}$ and $E_{2}$; shear moduli, $G_{12}$ and $G_{23}$; and Poisson’s ratios, $\nu_{12}$ and $\nu_{23}$. For calculating these engineering parameters, an inverse analysis is employed. Thus, the FEM simulations are carried out for three different cases, namely: the fiber orientation of (a) 0°, (b) 90°, and (c) 45°, respectively.

The engineering parameters by means of the FEM simulations are determined as follows:

1. For calculating effective longitudinal elasticity modulus, $E_{1}$, and Poisson’s ratio, $\nu_{12}$, at mesoscale, FEM simulations are performed on the CAD models with 0° fiber orientation. Linear displacement along the tensile direction, leading to constant strain for the unit length, ${\tilde{\epsilon }}_{XX}$ = 0.2 are expected. On the clamping side, X=0, Dirichlet boundary condition, $u_{X}=0$ is applied, and on the other end, X=2.5, is set to $u_{X}=0.5$. Then, strain energy is computed by $U=\frac{1}{2}\int \sigma_{ij}\varepsilon_{ij}dV$. Elasticity modulus, $E_{1}$ is determined by the reformulation of the strain energy equation by $E_{1}=\frac{U}{0.5{\tilde{\epsilon }}_{XX}^{2}V}$. By means of these simulations, ${\tilde{\epsilon }}_{YY}$ is calculated by evaluating the transverse contraction of the specimens. This leads to computation of Poisson’s ratio, $\nu_{12}$, by $\nu_{12}=-\frac{{\tilde{\epsilon }}_{XX}}{{\tilde{\epsilon }}_{YY}}.$
2. For determining effective transverse elasticity modulus, $E_{2}$, and Poisson’s ratio, $\nu_{23}$, at mesoscale, FEM simulations are carried out on the CAD models, where the fibers are oriented in 90°. Analogously, constant strain for the unit length ${\tilde{\epsilon }}_{XX}$ = 0.2 is assumed. On the mounting side, X=0, the specimens are clamped by Dirichlet boundary condition, $u_{X}=0$, and on the other side of the specimen, X=2.5, it is set to $u_{X}=0.5$. Elasticity modulus, $E_{2}$, is computed by the reformulation of strain energy equation, $E_{2}=\frac{U}{0.5{\tilde{\epsilon }}_{XX}^{2}V}$. ${\tilde{\epsilon }}_{ZZ}$ is analogously measured by these simulations and, hence, Poisson’s ratio, $\nu_{23}$, is determined by $\nu_{23}=-\frac{{\tilde{\epsilon }}_{YY}}{{\tilde{\epsilon }}_{ZZ}}.$ Furthermore, shear modulus, $G_{23}$, is calculated from $G_{23}=\frac{2(1+\nu_{23})}{E_{2}}.$
3. One more elasticity modulus is necessary for determining the $G_{12}$. FEM simulations are employed on the CAD models with 45° to make simplifications in the trigonometric part of the equations which is required for obtaining $G_{12}$. For more details about the simplifications, we refer to [33]. Constant strain for the unit length ${\tilde{\epsilon }}_{XX}$ = 0.2 is assumed analogously in these simulations. Again, at clamping side, X=0, Dirichlet boundary condition, $u_{X}=0$, is utilized, and on the other side, X=2.5, it is set to $u_{X}=0.5$. The elasticity modulus, $\bar{E}$, is analogously calculated by the reformulation of strain energy equation $\bar{E}=\frac{U}{0.5{\tilde{\epsilon }}_{XX}^{2}V}$. Then, shear modulus, $G_{12}$, is determined from
  (8) $$G_{12}=\frac{1}{\frac{4}{\bar{E}}-\frac{1}{E_{1}}-\frac{1}{E_{2}}+\frac{2\nu_{12}}{E_{1}}}.$$

**Figure 24:**
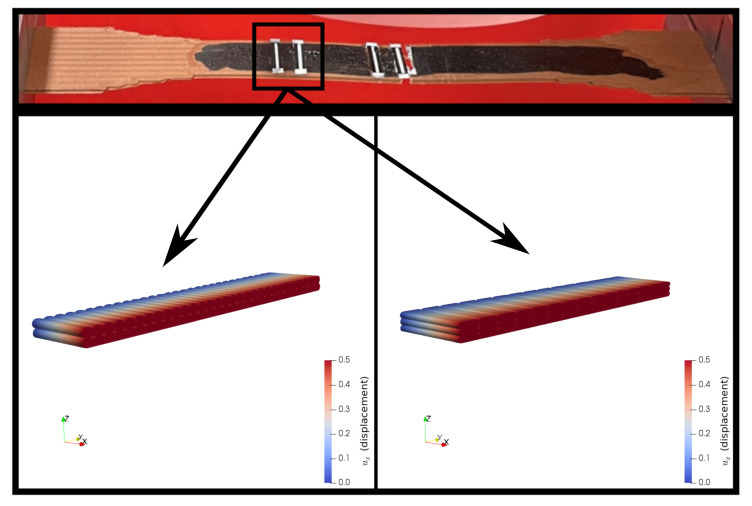
FEM simulations for uniaxial tensile tests of 0.3 mm thickness–0.4 mm raster width (**left**) and 0.2 mm thickness–0.8 mm raster width (**right**). One end of the specimens is clamped by Dirichlet boundary conditions, and the other end is set to $u_{X}=0.5$.

We provide Table 9 for all $E_{1}$ results. The differences between the elasticity modulus by the experiments and the computations are less than 6%, which validates the FEM simulations. The elasticity modulus, $E_{1}$, is increased with a lower layer thickness and greater raster width, similar to the outcomes of the experimental characterizations. An example uniaxial tensile test simulation by FEM is illustrated in Figure 24.

### 4.4. Multiscale Simulations

In the multiscale simulations, the computed stiffness matrix from the asymptotic homogenizations on the microscale is used as the input parameter in the computational homogenization on the mesoscale. These computations are analogously performed in the Python language with the open-source packages of the FEniCS project [57,58]. The multiscale homogenized effective parameters and, thereby, its stiffness matrix for the 0.3 mm layer thickness and 0.4 mm raster width in Voigt notation reads,

(9) $$C=\left[\begin{array}{cccccc} 5292.47 & 1881.55 & 1881.55 & 0 & 0 & 0\\ 1881.55 & 3962.02 & 1622.99 & 0 & 0 & 0\\ 1881.55 & 1622.99 & 3962.02 & 0 & 0 & 0\\ 0 & 0 & 0 & 1169.51 & 0 & 0\\ 0 & 0 & 0 & 0 & 1214.25 & 0\\ 0 & 0 & 0 & 0 & 0 & 1214.25 \end{array}\right].$$

We observe that the multiscale stiffness matrix is transverse isotropic as it is resulted by homogenized material models from microscale and mesoscale computations. The suggested representative volume elements (RVEs) on the microscale lead to isotropic elastic properties in the homogenized length scale. The unidirectional FDM printing method, which we carried out in the experiments, is the main reason for the transverse isotropic characteristics on the mesoscale.

**Figure 25:**
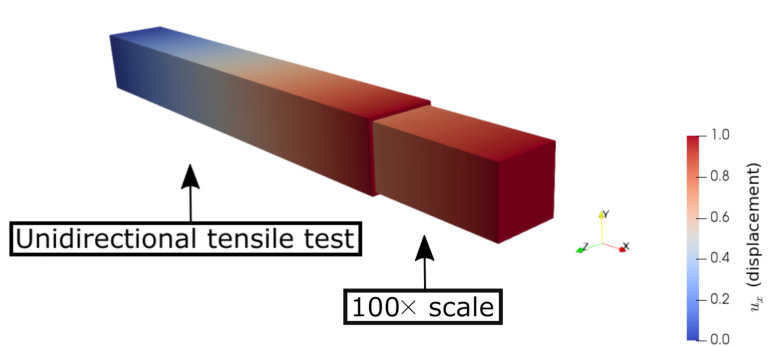
FEM simulation on a simple bar (100 × 100 × 750 mm) with the input parameter of the multiscale homogenized material model, Equation (9). The 100× scale of this simulation is illustrated in order to better visualize the transverse contractions along the *y* axis and *z* axis due to elongation (tensile direction) in the *x* axis.

The multiscale homogenized stiffness matrix incorporates the effects of the printing conditions, filament characteristics from the mesoscale, and the properties as well as the orientation of the reinforcement materials embedded in the matrix materials through the representative volume elements (RVEs). The stiffness matrix is also important data for the classical laminate theory (CLT). All stacking options can be found by using the rotation matrix, tensor mathematics, and Equation (9). We perform a unidirectional tensile test simulation with Equation (9) as an input parameter to be able to show the accuracy of the multiscale homogenization approach in this study, depicted in Figure 25.

## 5. Correlation between Experiments and Simulations

**Figure 26:**
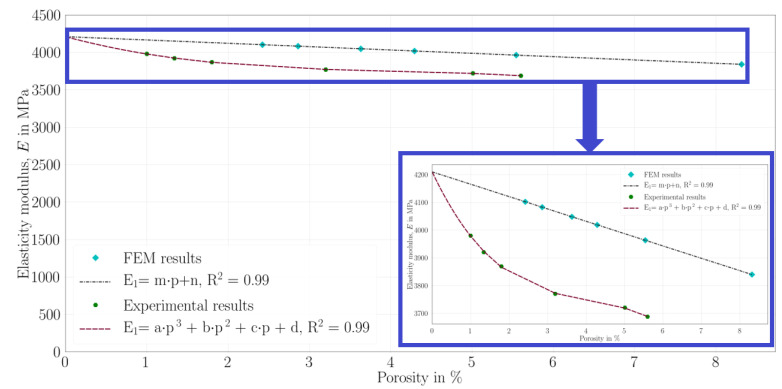
Correlation between elasticity modulus, $E_{1}$, and the porosity ratio calculated from manufactured specimens as well as from CAD images.

By means of the mechanical and optical characterizations as well as the FEM simulations, we find out that the mechanical properties of the FDM copper-reinforced PLA are quite correlative with the porosity ratio. Therefore, we build correlations between the elasticity modulus, $E_{1}$, and the porosity ratio for the manufactured specimens as well as the simulation results regardless of their process parameter information, namely the layer thickness and raster width. The aforementioned relation diagram is depicted in Figure 26. We emphasize that we initially compare the results of the mesoscale homogenization as the FEM simulation results because their input values are obtained by the filament manufacturer. The multiscale homogenization results are discussed in the following.

In Figure 26, $E_{1}$ and *p* are the elasticity modulus and porosity, respectively. The mesoscale FEM results have a quite linear porosity–elasticity modulus relation in Figure 26. A curve fit software is prepared and employed to the results in the Python language by means of the open-source packages of the LMFIT [60], which is based on nonlinear least squares minimization. The resulted equation and the parameters are obtained,

(10) $$E_{1}=m\times p+n,\;m\;=\;-44.516,\;n\;=\;4210,\;R^{2}\;=\;0.9999,$$

where $R^{2}=0.9999$ and $R_{adj}^{2}=0.9999$ show a proper fit to the mesoscale FEM results. The experimental results indicate that they have a quite nonlinear porosity–elasticity modulus relationship. Therefore, we apply a polynomial fit with 3rd grade. An analogous curve fit software in the Python language is utilized with the LMFIT algorithm, and the fit parameters are as follows

(11) $$E_{1}=a\times p^{3}+b\times p^{2}+c\times p+d,\;a\;=\;-5.4022,\;b\;=\;65.754,\;c\;=\;-291.85,\;d\;=\;4210,\;R^{2}\;=\;0.9995,$$

where the fit outcomes, $R^{2}=0.9995$ and $R_{adj}^{2}=0.9987$, indicate a suitable fit for the experimental results. It is clear that the simulations and experimental characterizations have different tendencies by decreasing the porosity. We interpret that the main reasons are the molecular effect and the production method employed in FDM.

On the mesoscale simulations, we model every detail of the geometries, all homogeneous, such as the fibers, contact areas, as well as the bottom surface contacting with the FDM build plate. On the contrary, this homogeneity does not exist in manufactured specimens due to a lack of molecular diffusion. We applied a 60 °C bed temperature, which means the bottom layers and the neighboring areas experienced some higher temperature effects than the top areas. Therefore, it may be expected that more molecular diffusions occur in the bottom parts, which results in a heterogeneous structure.

Due to the process technique employed in FDM, many different areas (solidified fibers, contacts, and the microporosities between them) occur in the structure (see Figure 15). Molecules are diffused along contact lines; hence, bonding occurs between deposited molten polymers and already solidifying ones. Sufficient molecular diffusions in contact lines are of importance because the FDM polymers have brittle behavior when they have weak bonding [51]. Many factors affect the molecular diffusion in the aforementioned areas, such as the temperature and the size of the contacts. A greater contact area is needed for bond formation [33,61], but this is not always a guarantee if there is not enough temperature effect.

In this study, we did not set any overlap between the fibers; all the specimens were FDM printed with 0% overlap in order to clearly investigate the effect of the layer thickness and raster width. However, bondings occur between filaments and layers due to the temperature and other process conditions, as seen in Figure 15. Furthermore, the porosity is decreased with a higher raster width and a lower layer thickness. Hence, greater contact areas occur in the structure and lead to a nonlinear increase in the elasticity modulus in Figure 26. Nevertheless, this nonlinearity does not exist in the FEM curve (see in Figure 26) because there was no molecular effect in the simulations.

In order to analyze the difference between the simulations and the experimental results, a detailed comparison is needed. Initially, three groups with similar porosity ratios from the experimental and mesoscale FEM simulation results are examined. The comparison is provided in Table 10.

After that, the elasticity modulus is computed by using the fit functions Equation (11) and Equation (10) for 1–6% porosity ratios. In this way, the elasticity modulus for the same porosities is compared in Table 11.

Ultimately, we compare all the experimental, FEM mesoscale, as well as the FEM multiscale simulation results in Table 12.

There are admissible differences between the mesoscale FEM simulations and the experimental results (lower than ∼ 7%). The reason is the aforementioned molecular effect in the manufactured specimens and the structural heterogeneity due to the process technique employed in FDM. A change in the process parameters also affects this heterogeneity, verified by different relative errors in Table 12.

The multiscale FEM results have some higher relative errors. We use the microscale homogenizations as the input parameters for the multiscale; there is a (4.6%) difference between the elasticity moduli of the filament (obtained from the filament manufacturer) and the homogenized microscale material properties. Because the FEM simulations are performed by using idealized CAD models, they may exhibit higher mechanical results. The simulations are carried out by assuming perfect bonding between the copper and PLA matrix, which is mostly overestimated. Moreover, the filament manufacturer uses some process additives and compatibilizers during filament extrusion, which are not studied herein. By using more detailed representative volume elements on the microscale, the difference between the experimental and FEM multiscale results possibly decreases. Multiscale homogenization is also very useful if the anisotropic effects of the microscale are needed to be investigated. In this way, material anisotropy (complex filler shapes and their orientations in the matrix) can be incorporated into the mesoscale effects (such as different layer layups).

In this study, we observed only transverse isotropy after multiscale homogenization (see Equation (9)). Although we determined 20.4 volume % copper particles in the filament, they had spherical shapes, and therefore, the material exhibits isotropic behavior on the microscale. This is verified by the relative low error between the experimental and mesoscale FEM results because we model the microscale as an isotropic linear elastic material in these simulations. Transverse isotropy derives due to the unidirectional printing order of the specimens, which we included through computational homogenization on the mesoscale.

We suggest the mesoscale FEM is suitable if only polymer filaments are used for the FDM printing. However, if composite materials are FDM printed, especially filaments with anisotropic particles, multiscale FEM simulations are needed for a comprehensive analysis.

## 6. Conclusions

In this study, we examined the effect of different FDM process parameters, namely layer thickness and raster width. Mechanical and optical characterizations were performed. By means of optical characterizations, the micro- and mesostructures were investigated. In this way, multiscale homogenization was carried out through FEM simulations. The porosity–elasticity modulus relationship of the FDM copper-reinforced PLA was discussed for the experiments as well as the simulations.

- We FDM printed specimens with different layer thicknesses (0.2 and 0.3 mm) as well as three different raster widths (0.4, 0.8, and 0.96 mm). We conducted tensile tests with a laser extensometer and a high-resolution camera (4272×2848 pixels, 1 picture/2 s). By means of the tensile characterizations, we observed that the elasticity modulus and the ultimate tensile strength increased by using a lower layer thickness and a greater raster width for FDM-printed copper-reinforced PLA composites. The deformations of the specimens during the tensile tests were examined by a high-resolution camera in detail.
- We investigated how the mesostructure was altered with different process parameters. By means of our digital image correlation code with its machine learning algorithm, we calculated the porosity ratios for each group of specimens. The porosity ratio decreased with a lower layer thickness and a greater raster width.
- Scanning electron microscopy (SEM) and energy dispersive X-ray spectroscopy (EDS) characterizations were carried out, and we analyzed the microstructure of the specimens with secondary electron and back-scattered electron modes. Through the EDS, we differentiated the materials from the SEM analysis in different images, such as carbon and copper.
- We prepared two representative volume elements for the microscale by considering the volume % and the dimensions of the copper particles. We carried out asymptotic homogenization with periodic boundary conditions, and we obtained the stiffness matrix for each of the RVEs. Finally, we compared the $E_{1}$ from their stiffness matrix and the elasticity modulus from the filament and found an admissible difference (4.6%) between them.
- We prepared CAD models for the mesoscale simulations by regarding the porosity ratio and the mesostructural features obtained from the optical microscopy analysis. Computational homogenization by means of the FEM simulations was carried out. We determined the (lower than ∼7 %) relative error between the simulations and the experimental results.
- We correlated the porosity ratios and elasticity modulus for both the manufactured specimens and the mesoscale FEM simulations. We found out that there was a nonlinear dependence for the experimental results. A lower porosity and hence greater contacts with adjacent fiber layers increased the molecular diffusion, which led to a stronger bond formation in the structure. This resulted in a higher elasticity modulus and a nonlinear increase with porosity. As we excluded the molecular effects in the simulations and modeled the whole structure homogeneously, there was a linear dependency for the simulations.
- We performed a uniaxial tensile test simulation on a simple bar with the multiscale homogenized stiffness matrix as an input parameter. In this way, we checked and validated the proposed homogenization system. By comparing the simulation, its 100× scale (see Figure 25), and the laboratory experiments (see Figure 13), we observed that similar elongation and contraction profiles of the manufactured specimens occurred in the simulations (elongation in the *x* axis and transverse contractions along the *y* and *z* axes, Figure 25).

## Figures and Tables

**Table 1:** Process parameters of FDM printing.

Parameter	Value	Unit
Print speed	55	mm/s
Initial layer speed	20	mm/s
Print acceleration	4000	mm/s${}^{2}$
Print temperature	225	°C
Print temperature initial layer	225	°C
Final printing temperature	215	°C
Bed temperature	60	°C

**Table 2:** Material properties of copper-reinforced PLA from the manufacturer, PrimaCreator (Malmö, Sweden).

	Value	Unit	Method
Specific gravity	3.41	g/cc	SO 1183
Elongation at break	4.5%	-	ISO 527
Yield stress	18.3	MPa	ISO 527
Tensile modulus	4210	MPa	ISO 527
Melting point	±195	°C	ISO 294
Vicat softening temp.	±65	°C	ISO 306

**Table 3:** Experimental design with three raster widths and two layer thicknesses. All groups are FDM printed and tested on five specimens for obtaining a statistical confidence interval.

Layer Thickness/Raster Width	0.4 mm	0.8 mm	0.96 mm
0.2 mm	5 specimens	5 specimens	5 specimens
0.3 mm	5 specimens	5 specimens	5 specimens

**Table 4:** Specimen specifications in mm.

Thickness	Angle	a	b	c	d	e
0.6	R60	150	60	21.4	21.7	12

**Table 5:** Elasticity modulus and ultimate tensile strength from experimental characterizations: mean value of tested specimens, for the test group, see Table 3.

Elasticity Modulus (MPa) Ultimate Tensile Strength (MPa)			
**Layer Thickness/Raster Width**	**0.4 mm**	**0.8 mm**	**0.96 mm**
0.3 mm	3688.31 11.02	3770.46 11.34	3869.50 11.63
0.2 mm	3720.77 11.17	3920.40 11.81	3980.12 12.05

**Table 6:** Porosity quantification obtained from the microscope images.

Porosity Ratio			
**Layer Thickness/Raster Width**	**0.4 mm**	**0.8 mm**	**0.96 mm**
0.3 mm	5.60%	3.20%	1.80%
0.2 mm	5.01%	1.34%	1.00%

**Table 7:** Porosity calculated from the CAD models prepared by using the ideal inner structures.

Porosity Ratio			
**Layer Thickness/Raster Width**	**0.4 mm**	**0.8 mm**	**0.96 mm**
0.3 mm	8.31%	4.29%	3.63%
0.2 mm	5.54%	2.86%	2.42%

**Table 8:** Elasticity moduli, $E_{1}$, calculated from homogenized stiffness matrix and filament.

Analysis	Elasticity Modulus, $E_{1}$
RVE1	4413.5 MPa
RVE2	4412.7 MPa
Filament	4210.0 MPa

**Table 9:** Elasticity moduli, $E_{1}$, determined by the FEM simulations on the CAD models with 0° fiber orientation.

Elasticity Modulus			
**Layer Thickness/Raster Width**	**0.4 mm**	**0.8 mm**	**0.96 mm**
0.3 mm	3839 MPa	4018 MPa	4048 MPa
0.2 mm	3963 MPa	4082 MPa	4102 MPa

**Table 10:** Relative error of elasticity modulus between similar porosities from three experimental and FEM groups.

Experimental Porosity (%)	FEM Porosity (%)	Relative Error (%)
3.63	3.2	3.079
4.29	5.01	4.858
5.54	5.6	3.695

**Table 11:** Comparison of estimated elasticity moduli ($E_{1}$) computed by curve fit equations.

Porosity	Elasticity Modulus (MPa)		Relative Error (%)
	Experimental Results	FEM Simulations	
1.0	3978.50	4165.48	4.48
1.5	3901.93	4143.22	5.82
2.0	3846.09	4120.96	6.67
2.5	3806.92	4098.71	7.11
3.0	3780.37	4076.45	7.26
3.5	3762.39	4054.19	7.19
4.0	3748.92	4031.93	7.01
4.5	3735.91	4009.67	6.82
5.0	3719.32	3987.42	6.72
5.5	3695.09	3965.16	6.81
6.0	3659.16	3942.90	7.19

**Table 12:** Elasticity moduli ($E_{1}$) from FEM mesoscale and multiscale simulations as well as experimental results and their relative errors. We represent layer thickness and raster width of the specimens by h and w, respectively.

Test Groups	Elasticity Modulus (MPa)			Relative Error (%) (Experiments vs. FEM Mesoscale)	Relative Error (%) (Experiments vs. FEM Multiscale)
	Experiments	FEM (Mesoscale)	FEM (Multiscale)		
h = 0.2 mm w = 0.96 mm	3980.12	4102.30	4300.64	2.97	7.45
h = 0.2 mm w = 0.8 mm	3920.40	4082.78	4280.18	3.97	8.40
h = 0.3 mm w = 0.96 mm	3869.50	4048.29	4244.02	4.41	8.82
h = 0.3 mm w = 0.8 mm	3770.46	4018.97	4213.28	6.18	10.50
h = 0.2 mm w = 0.4 mm	3720.77	3963.52	4155.15	6.12	10.45
h = 0.3 mm w = 0.4 mm	3687.99	3839.91	4025.56	3.95	8.38

## Data Availability

Raw data were generated at the TU Berlin, Institute of Material Science and Technology, as well as the Institute of Mechanics. Derived data supporting the findings of this study are available from the corresponding author (A.Ö.) upon request.
